# Evolution of proteins and proteomes: a phylogenetics approach

**Published:** 2007-02-24

**Authors:** Toni Gabaldón

**Affiliations:** Bioinformatics Department, Centro de Investigación Principe Felipe

**Keywords:** protein evolution, proteome evolution, protein domains, phylogenetics, ancestral sequence reconstruction, function prediction

## Abstract

The study of evolutionary relationships among protein sequences was one of the first applications of bioinformatics. Since then, and accompanying the wealth of biological data produced by genome sequencing and other high-throughput techniques, the use of bioinformatics in general and phylogenetics in particular has been gaining ground in the study of protein and proteome evolution. Nowadays, the use of phylogenetics is instrumental not only to infer the evolutionary relationships among species and their genome sequences, but also to reconstruct ancestral states of proteins and proteomes and hence trace the paths followed by evolution. Here I survey recent progress in the elucidation of mechanisms of protein and proteome evolution in which phylogenetics has played a determinant role.

## Introduction

During the 1960s, a decade before DNA sequencing become feasible, biochemists and molecular biologists were increasingly attracted by questions regarding the evolution of proteins. By that time, protein sequencing techniques were producing a growing number of sequences (Eck and Dayhoff 1966), and soon it was realized that proteins and nucleic acids could be used to document the history of past evolutionary events (Zuckerkandl and Pauling 1965). Computers, which had already been used in the sequence determination of proteins (Dayhoff 1965), were also recruited to the task of comparing sequences from different organisms. In their pioneering work, Fitch and Margoliash (1967) computationally compared sequences of the respiratory pigment cytochrome c from different organisms to assess their phylogenetic relationships. Since then, bioinformatics has been increasingly involved in protein evolution (Hagen 2000). Bioinformatics applications to the study of protein evolution include, among many others, algorithms to efficiently align similar sequences, to detect homologous sequences in large databases or to reconstruct phylogenetic trees from a given set of sequences.

The relationship between bioinformatics and the study of protein evolution was further strengthened with the advent of large-scale sequencing projects. The growing number of sequences stored in the databases, including those of complete genomes, provided a completely new dimension to the study of protein evolution: that of the evolution of complete proteomes. New bioinformatics tools were developed that allowed the comparison of complete genomes, the efficient detection of orthology relationships and the reconstruction of the evolution of complete proteomes. Almost four decades after the first computer-aided evolutionary analysis of proteins, there is a vast scientific literature reporting bioinformatics analyses that focus on protein or proteome evolution. The aim of this review is to provide a general overview of recent bioinformatics approaches to the study of the evolution of proteins and proteomes that involve the use of phylogenetics. Due to space limitations, I will focus on the evolution of proteins at the sequence level and of proteomes in terms of their protein content. Therefore, I purposely excluded considerations regarding evolution of protein structures, protein interaction or regulatory networks. I start providing an overview of the different bioinformatics applications that can be used to reconstruct the phylogenetic relationships of a protein family to then describe its applications. These include the reconstruction of ancestral protein sequences, the determination of orthology and paralogy relationships and the use of phylogenetic profiles and co-evolution to predict protein function. Subsequently, the evolution of proteins is considered within the context of the pathways and the complete proteomes in which they function. To illustrate the different sections, some specific examples from the literature have been selected.

## Phylogenetics and the study of protein evolution

**Figure 1:**
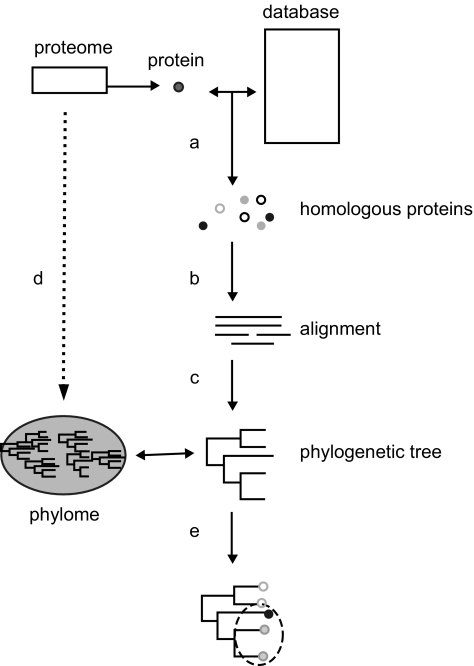
Schematic representation of the phylogenetic analysis process of a protein family. The protein sequence of interest is compared to a sequence database to retrieve significantly similar proteins (a); homologous proteins are aligned to place homologous residues on top of each other (b); under the assumption of an evolutionary model, a phylogenetic tree representing the evolutionary relationships among the protein sequences is reconstructed (c); if this process (a to c) is repeated over all proteins encoded by a genome, the total set of phylogenetic trees or phylome is reconstructed (d); the topology of the phylogenetic tree can be subsequently analyzed for different purposes, e.g to determine orthology and paralogy relationships (e).

The phylogenetic analysis of a protein (Figure 1) starts with the detection of other members of its family. This is usually done by comparing the sequence of the protein of interest with other sequences stored in the databases and, subsequently, selecting the hits that are significantly similar. The assumption is that proteins with similar sequences are derived from a common ancestral protein. In other words, they are considered to be homologous proteins (Fitch 1970, 2000). Several algorithms have been developed that allow efficient automatic detection of homologous proteins in large databases. These include pair-wise comparison algorithms like Smith-Waterman (Smith and Waterman 1981) and its faster approximation BLAST (Altschul, et al 1997). The sensitivity of such homology searches has been more than doubled (Park, et al 1998) by profile-based methods such as PSI-BLAST (Altschul and Koonin 1998, Altschul, et al 1997) or hidden markov models (Baldi, et al 1994, Eddy 1998). Once the sequences of a protein family are retrieved, they can be aligned. The alignment of multiple sequences basically aims to place ‘homologous’ residues of different proteins on top of each other. This constitutes a crucial step for the study of the evolution of proteins because it is assumed that all positions in a column of a multiple sequence alignment derive from a common ancestral residue. Several multiple-sequence alignment algorithms exist that combine speed with reasonable accuracy, these include programs such as ClustalW (Thompson, et al 1994), T-Coffee (Notredame, et al 2000) or MUSCLE (Edgar 2004).

By applying a specific evolutionary model to explain the amino acid substitutions observed in the multiple sequence alignment, the evolutionary distances between all pairs of proteins can be computed. This evolutionary distance, which reflects the expected mean number of changes per site that have occurred since two sequences diverged from their common ancestor, is used by the so-called distance-methods for phylogenetic inference. One such method is Neighbor Joining (NJ). NJ constitutes a good and fast heuristic algorithm that estimates the “minimal evolution” tree, a phylogenetic tree which minimizes the sum of the lengths (evolutionary distances) of all its branches (Saitou 1987). NJ and variations of it have long been proven to be quite efficient in finding the “right” tree topologies given a set of homologous sequences (Kuhner and Felsenstein 1994, Takahashi and Nei 2000), although its accuracy may suffer in large datasets (Nakhleh, et al 2002). Compared to other methods, NJ has the advantage of being very fast, which allows the construction of large trees including hundreds of sequences. Therefore, it is usually the method of choice when doing large-scale phylogenetic approaches.

A different approach for phylogenetic inference is that of Maximum Likelihood (ML) (Schadt, et al 1998). Here, the concept of likelihood refers to the probability that a certain tree with a set of parameters (topology, branch-lengths, etc) produces, assuming a specific evolutionary model, a given set of data (sequences). ML-methods try to find the tree with the maximal likelihood to produce the variation observed in the given set of data. However, computing the likelihood of all possible trees for a decent number of proteins is a very computationallyintense task and becomes unfeasible for large sets of sequences. Therefore, all practical methods rely on heuristics that are able to reduce the search-space and find good suboptimal trees in a reasonable time. For instance PhyML (Guindon and Gascuel 2003) uses a simple hillclimbing algorithm to optimize a seed NJ-tree whereas MrBayes (Ronquist and Huelsenbeck 2003) uses bayesian inference with a Markov Chain Monte Carlo (MCMC) algorithm. Recently, some ML methods have been developed that allow the joint iterative reconstruction of the protein alignment and the corresponding phylogenetic tree (Redelings and Suchard 2005). The phylogenetic reconstruction with such methods is usually improved by the implementation of models of sequence evolution that allow the substitution rates to vary among the different positions (Felsentsein 2001). These models, which generally approximate a Gamma distribution for the variation of rates across positions, better reflect the real situation in which functional constraints are not uniform over the entire protein sequence. Even the substitution rate for a given residue may vary over time, a process known as heterotachy, which is taken into account by recent implementations (López et al, 2002).

Yet another type of phylogenetic inference is that of Maximum Parsimony (MP) (Felsenstein 1996), which selects the tree that requires the minimum number of character changes (mutations) to explain the given set of sequences. MP approach does not allow the correction for multiple mutations per site and it is more prone to the so-called long-branch attraction effect (placing long branches preferentially together towards the root of the tree).

Perhaps, the development of algorithms for phylogenetic reconstruction, database search and multiple sequence alignment represents the most visible contribution of bioinformatics to the study of protein evolution. This is not surprising because the applications of phylogenetic trees are many and they are used in diverse fields. Besides their traditional taxonomic use for inferring the evolutionary relationship between organisms, phylogenetic trees can be used to establish orthology and paralogy relationships among proteins (Fitch 2000), to detect horizontal gene transfers (HGT) (Bapteste, et al 2004), gene and genome duplications (Van de Peer 2004), positively selected residues (Bielawski and Yang 2004), to define strains in epidemiologic studies, to predict functional interactions among coevolving genes (see bellow) or to estimate model parameters and substitution rates (Lio and Goldman 1998). Phylogenetics is thus central for many evolutionary analyses, some applications which include a phylogenetic reconstruction at some stage will be shown in more detail in the following sections.

## Orthology and paralogy considerations

Orthology and paralogy are key concepts in the field of protein and proteome evolution. The use of these terms has been extended as comparative and evolutionary genomics have penetrated other fields. However, there is still some confusion about their exact meanings. Many researchers believe that orthologous proteins are simply proteins with the same function in different organisms, whereas paralogs are simply homologs within one organism. These definitions do not agree with the original given by Walter Fitch (1970), in which orthologs are homologous genes (proteins) derived by speciacion from a common ancestor whereas paralogs are homologs derived by duplication. Therefore the definition of orthology and paralogy is strictly phylogenetic and do not include any functional consideration. The correct detection of orthology relationships allows the comparison of genomes in terms of their gene content, an essential step for studying the co-evolution of proteins and the evolution of complete proteomes (see bellow).

**Figure 2:**
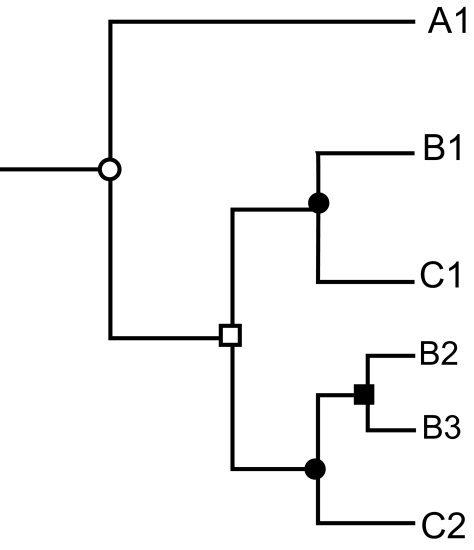
Orthology and paralogy relationships within a protein family. The phylogenetic tree of a hypothetical protein family comprising a total of six members: one in species A (A1), three in species B (B1, B2 and B3) and two in species C (C1, C2). Speciation and duplication events are represented as circles and squares, respectively. A first duplication event (white square) occurred before the B–C speciation (black circle) while a later one (black square) occurred within the B lineage. In this scenario the only protein present in A (A1) is orthologous to all the others and vice versa. When comparing members from B and C: C1 is orthologous to B1, while C2 has two orthologs in B (B2 and B3).

The detection of orthology relationships is ideally performed by detecting speciation and duplication events through phylogenetic analysis (Figure 2). Alternative methods which only rely on sequence similarity levels like “best bi-directional hits” (Huynen and Bork 1998) and its multiple-genome extensions (Tatusov, et al 2001), are more prone to errors, especially when there is variation in the rate of sequence evolution within an orthologous group (Eisen and Fraser 2003). Recent developments in the automatic detection of orthology from phylogenetic trees (Dufayard, et al 2005, Gabaldón and Huynen 2005) are promising and allow its application over large datasets.

## Protein domains as evolutionary units

We have seen how phylogenetic reconstruction can ascertain the evolutionary relationships within members of a protein family, which evolved independently after speciation and duplication events. A complication may arise when different protein families combine through recombination events. In this case, the evolutionary unit is not the full protein anymore but a smaller, discrete molecular entity called protein domain (Doolittle 1995).

**Figure 3:**
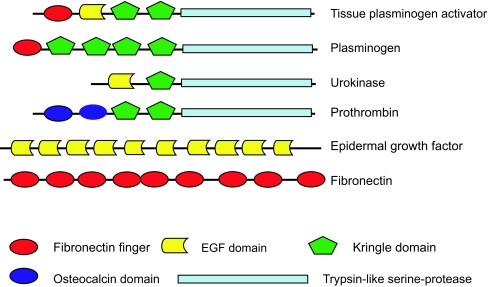
Schematic domain organization of tissue plasminogen activator protein (TPA) and several other proteins that share, at least, one of the domains present in TPA. The specific function of each protein emerges from a particular domain combination.

One of the first multi-domain proteins to be studied in detail was the tissue plasminogen activator (TPA), which contains four different domains that are also present in other protein families (figure 3). The fact that different parts of TPA presented homology with different protein families is an indication that TPA is actually an evolutionary chimera. Thus, protein domains of TPA are the evolutionary units that underwent duplication, followed by recombination with other domains, and in present-day proteins are found as units of various multi-domain arrangements. Initially, multi-domain proteins were considered an exception to the rule but, as more sequences became available in the databases more cases of mosaic proteins and different domain combinations were identified. With the current data it is estimated that multi-domain proteins comprise more than two-thirds of the proteins encoded by prokaryotic genomes (Teichmann, et al 1998), and even a greater fraction in eukaryotic genomes (Gerstein 1998).

Multi-domain proteins are created through gene duplication and recombination events. Since the role of protein domains is dependent on the context in which they are found, the emergence of new domain combinations may involve the creation of completely new functions. Not surprisingly, this mechanism of domain shuffling has been extensively exploited during evolution. This constitutes another example of how completely new functions can emerge from the tinkering of preexisting components.

Several large-scale studies have recently focused on the evolution of proteomes in terms of the domain combinations that they contain. One of such studies shows that the repertoire of domain combinations observed in nature is just a small fraction of all possible combinations (Apic, et al 2001). This suggests that domain combinations are subjected to strong selection during evolution. While most of the protein domains can be found in combination with just one or two different domains, some others, the so called promiscuous domains, can be combined with many different domains (Wuchty 2001). Interestingly, most protein domains are present in eukaryotes, bacteria and archaea. This suggests a very ancient origin for all of them and a last common universal ancestor possessing an almost complete protein domain repertoire. In contrast, most domain combinations are kingdom- or lineage-specific and have, therefore, appeared in later stages during evolution.

## Ancestral sequence reconstruction

In the phylogenetic tree, the internal nodes represent ancestral sequences from which proteins at the leaves have evolved. The properties of these sequences are often relevant to ascertain how modern functions come about or what mutations were crucial in the development of functional specificities within a protein family. Infer the properties of such ancestral sequences or even to reconstruct them is the aim of the emerging field of “ancestral sequence reconstruction”. Ancestral sequence reconstruction uses extant sequences and the phylogenetic relationships among them to infer the most plausible ancestral sequences (Cai, et al 2004, Yang, et al 1995). Attending their scope, ancestral reconstruction can be divided into joint reconstruction (Pupko, et al 2000), when it finds the most likely set of amino acids for all internal nodes at a site, or marginal reconstruction (Koshi and Goldstein 1996), when it limits the inference to a particular node or sets of nodes.

Once the sequence of interest is computationally reconstructed, it is possible to infer its functional properties based on the presence of specific residues at key positions, e.g. the active site. Additionally, one can take one step further and synthesize the ancestral protein to directly test its biochemical properties, an experiment that is often referred to as “ancestral sequence resurrection” (Thornton 2004). An interesting application of ancestral sequence reconstruction is the testing of specific scenarios that involve extinct organisms. For instance, knowledge about the light-response properties of the visual protein rhodopsin from early dinosaurs is useful in the assessment of their day and night habits. Using sequences from living vertebrates, Chang and colleagues reconstructed, and subsequently synthesized, the ancestral archosaurs rhodopsin (Chang, et al 2002). Its biochemical characterization suggested that these early dinosaurs could have seen well under dim lighting conditions. A few more examples include the reconstruction of ancestral hormone receptors (Thornton, et al 2003) and ancestral bacterial translational elongation (EF-Tu) proteins (Gaucher, et al 2003).

But how reliable are these reconstructions and therefore the functional inferences?This is a hotly debated issue and some researchers remain sceptic over conclusions on extinct sequences. In general, ancestral sequence reconstruction suffers from the same weaknesses as other evolutionary methods: its correctness depends on the quality of the data and the adequateness of the model. Moreover, these methods are based on probabilistic approaches and thus the reconstructed sequences are not free of ambiguities. With no real ancestral sequences at hand it is hard to judge the correctness of the reconstructions. Recently, some analyses have suggested that ancestral reconstruction may indeed have a sequence-compositional bias that can affect inference of ancestral function (Krishnan, et al 2004). A reasonable solution to these caveats is to base the functional inference not on a single reconstructed protein but on a sample of possible ancestral proteins, which takes into account ambiguously reconstructed positions. Nevertheless, the question of how many ancestral sequence samples are necessary to reliably estimate ancestral function remains open.

## Use of phylogenetic profiles to infer function

One of the most powerful techniques for assigning a biological function to a protein sequence is the detection of homologous sequences with known function in a sequence-similarity search. When performing this transfer of knowledge between similar sequences we are using an evolutionary approach: we assume that the sequences share a common ancestor and that their function has been maintained during evolution. However, homology-search is not the only way in which the evolutionary analysis of a protein can serve to infer its functional role.

**Figure 4:**
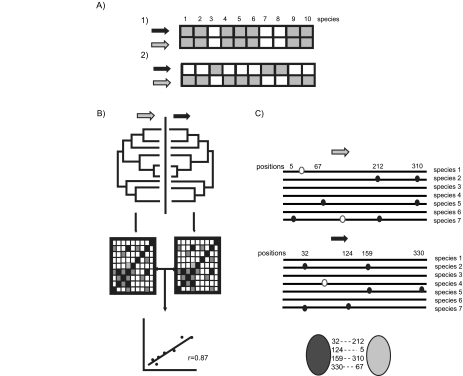
Co-evolution as a tool for functional inference. Several methods that use coevolution to predict functional interactions among two protein families (grey and black arrows) are illustrated. A) A similar pattern (1) of presence (grey boxes) and absence (white boxes) in a set of genomes might indicate that both proteins function in the same biological process or pathway. Complementary phylogenetic profiles (2) suggest a nonorthologous gene displacement event, i.e. both proteins perform the same function and can functionally replace each other. B) Similarity of phylogenetic trees, which can be measured by the correlation of the distance matrices as shown in the figure, are indicative of similar evolutionary constrains for both protein families. This, in turn, suggests that the two proteins interact physically. C) Correlated mutations (black circles), those that occur in both proteins in the same set of species, can be detected by the comparison of the protein alignments from the two protein families. The method predicts not only that the two proteins interact (ovals) but also which are the interacting residues (numbered residues).

In recent years, as part of the so-called genome-context analyses (Gabaldón and Huynen 2004), several methods have been developed that exploit the co-evolution of protein families to infer a functional interaction between them. One such techniques, called gene co-occurrence or phylogenetic profiles (Huynen, et al 1998, Pellegrini, et al 1999), compares the patterns of presence/absence of proteins in a set of complete genomes and predicts functional interactions between proteins with similar profiles (figure 4-A). This method is based on the observation that proteins with a similar distribution across species have a high tendency to functionally interact (Gaasterland and Ragan 1998, Huynen and Bork 1998, Huynen and Snel 2000, Pellegrini, et al 1999). It must be noted, however, that the reverse assumption, that functionally interacting proteins have a similar distribution accross species, is not necessarily true as we will see in the following section. Moreover, the detection of proteins with complementary phylogenetic patterns might indicate a non-orthologous gene displacement and thus a similar function for both protein families (Galperin and Koonin 2000).

Another variant of the use of co-evolution to predict protein function uses the evolutionary information that is contained in the sequences (figure 4-B). For specific protein families that are known to physically interact, such as the chemokinereceptor system (Goh, et al 2000, Hughes and Yeager 1999), it was shown that their phylogenies are more similar to each other than expected considering the evolutionary divergence between the species. This suggested the existence of correlated evolution reflecting similar evolutionary constraints. Some authors (Pazos and Valencia 2001, Valencia and Pazos 2003) have applied this property to predict interaction partners in *E. coli* by detecting significantly correlated positions between the distance matrices used to build the phylogenetic trees. Others (Ramani and Marcotte 2003) used a similar approach to predict the binding specificities among members of 18 ligand and receptor families with many members in the human genome. Finally, the co-evolution of interacting proteins can be analyzed more closely by searching for amino acid substitutions that are correlated in both protein families (they occur in the same species) (figure 4-C). These positions may correspond to residues on the interface that undergo mutations in one protein to compensate the effects of mutations in the other (Dimmic et al 2005, Fukami-Kobayashi et al 2002, Pazos, et al 1997, Pazos and Valencia 2002). This method has the advantage of predicting not only the interacting proteins, but also the residues potentially involved in the interaction. With more fully sequenced genomes to come, the accuracy and coverage of these genome-context techniques can only improve. It is also expected that new discoveries of mechanisms of protein and proteome evolution will fuel the development of new techniques that exploit them to predict protein function.

## Phylogenetic diversity of protein complexes and pathways

Proteins do not work as isolated entities. Instead, they perform their function through interactions with other proteins, as part of pathways, complexes and other types of functional modules. Therefore, to fully understand the evolution of a protein it is necessary to consider it in the context of the evolution of its functional partners. Recently, the availability of fully sequenced genomes has enabled the comparison across species of the composition of pathways, protein complexes and other functional modules (Huynen, et al 2005). Such comparative analyses are usually based in the detection of orthology relationships between the components of a functional system in a certain species and proteins encoded in other genomes. Perhaps the most unexpected result from the first analyses of this kind was the finding of a relatively large degree of variation across species in the composition of metabolic pathways and complexes. In the case of large complexes, comparative genomics analyses have revealed significant variations of the subunit content in, among others, the proteasome (Gille, et al 2003), the nuclear pore complex (Mans, et al 2004) and the eukaryotic NADH:ubiquinone oxidoreductase (Complex I) (Gabaldón, et al 2005). In the latter, the differences were mapped onto the species phylogeny and, assuming a parsimonious scenario, the history of gain and loss of subunits was reconstructed. The results showed a non-modular evolution of Complex I in the eukaryotes that contrasts with the modular pattern of evolution observed for this complex in the prokaryotes (Friedrich and Weiss 1997).

In the case of pathways, the evolutionary analyses of glycolysis (Dandekar, et al 1999), citric acid cycle (Huynen, et al 1999) or tryptophan synthesis (Xie, et al 2003), also revealed large divergences from the canonical pathway topology described in the textbooks. These results are indicative of a lack of modularity in the evolution of biochemical pathways. In other words, biochemical pathways do not seem to constitute evolutionary units that are either completely present or completely absent from a certain organism. Instead, a wide range of intermediate incomplete states of the pathway can be found. One might argue, however, that the lack of observed modularity might be related to the fact that the splitting of the cell metabolism into different pathways is rather artificial. For instance, glycolysis has many entry and exit points that are connected to other pathways such as the pentose phosphate pathway, glycerolipid metabolism or fructose and mannose metabolism. Therefore, the presence of pathways connected to glycolysis might involve the presence of glycolytic enzymes even in the abscence of glycolysis itself.

To overcome such conceptual issues, Snel and Huynen (Snel and Huynen 2004) performed a large-scale analysis of the variation of functional modules that were defined using various criteria, including the automatic inference from high-throughput experiments results. Their observations are consistent with a general low degree of modularity in the evolution of functional modules. Nevertheless, half of the functional modules do tend to evolve more cohesively than random, indicating that a certain level of evolutionary coherence exists between functionally interacting proteins.

## Phylogenetic reconstruction of ancestral proteomes

In the genomic era it has been possible to move from the evolutionary analysis of single protein families to that of complete genomes and proteomes. Large-scale comparative genomics analyses have shown that, during evolution, the protein repertoire encoded in a species genome is continuously shaped by processes such as gene loss, gene gain and gene duplication. These processes can be studied and quantified by reconstructing ancestral proteomic states along the species tree (Snel, et al 2002). Moreover, as in the case of ancestral sequence reconstruction, reconstruction of ancestral proteomes allows us to test specific evolutionary scenarios.

One of the first such scenarios to be studied with the help of comparative genomics was the origin of the first cell and the properties of the so-called Last Universal Common Ancestor (Kyrpides, et al 1999, Mushegian and Koonin 1996). The reconstruction of this ancestral proteome involved the comparison of fully-sequenced genomes in terms of their content in protein-coding genes and a parsimonious reconstruction of the ancestral protein content. Recent estimates, that correct for horizontal gene transfers and non-orthologous gene displacements, suggest a simple last universal common ancestor with only 500–600 proteins (Koonin 2003). Although that amount of proteins might seem very small, it represents a substantial complexity if we consider that the minimal proteomic set to sustain cellular life in a rich medium could comprise as few as 206 proteins (Gil, et al 2004).

Another evolutionary scenario that has been investigated through ancestral proteome reconstruction is that of the origin of mitochondria. Mitochondria are eukaryotic organelles that originated from the endosymbiosis of an αproteobacterium and a proto-eukaryotic cell (Gray, et al 1999). Several hypotheses have been proposed that explain the initial endosymbiosis in terms of different metabolic properties of the host and the endosymbiont (Martin, et al 2001). To address this issue Gabaldón and Huynen reconstructed the mitochondrial ancestor proteome and inferred its metabolism (Gabaldón and Huynen 2003). In this case a phylogenomic approach was used to reconstruct the ancestral proteome. First, thousands of phylogenetic trees were reconstructed to subsequently select those whose topology indicated the presence of a member of that protein family in the mitochondrial ancestor. In addition, by mapping onto metabolic maps the functions of those protein families, the proto-mitochondrial metabolism was inferred. The emerging picture is that of a (facultatively) aerobic endosymbiont catabolyzing compounds provided by the host. In the absence of a reconstructed proteome for the host, it is difficult to define a specific symbiotic scenario. Nevertheless, the conservation of a diverse set of protomitochondrial pathways in the modern eukaryotes suggests a multifaceted benefit for the host cell.

## Concluding remarks

The advent of bioinformatics, in combination with the availability of data obtained at a genome scale, has radically changed the way in which we study protein and proteome evolution. First, sophisticated tools for the comparison of protein sequences and the reconstruction of phylogenetic trees have allowed a better understanding of protein evolution at the molecular level. Moreover, the large variation observed in the composition of functional modules and proteomes from different species shows the great plasticity of living systems to adapt to different environments. Finally, through reconstructions of ancestral states it is now possible to trace the series of events that have shaped proteins and proteomes. Even the resurrection of extinct molecules is now a possibility. Will we see one day the experimental resurrection of an extinct cellular organism based on an ancestral proteome reconstruction? We will probably have to wait a long while. What is certain, however, is that future developments in bioinformatics will continue to shed light on the underlying mechanisms that govern the evolution of proteins and proteomes.
